# Frühe Quellen eines Sexualmediziners aus urologischer Sicht: Max Marcuse 1877–1963: „Arzt für Haut- und Harnleiden“

**DOI:** 10.1007/s00120-023-02176-4

**Published:** 2023-09-01

**Authors:** Friedrich H. Moll, Florian G. Mildenberger

**Affiliations:** 1 Institut für Geschichte, Theorie und Ethik der Medizin, Universität Düsseldorf, Düsseldorf, Deutschland; 2 Museum, Bibliothek und Archiv zur Geschichte der Urologie, Deutsche Gesellschaft für Urologie e. V., Düsseldorf Berlin, Deutschland; 3 Urologische Klinik, Kliniken der Stadt Köln GmbH, Neufelder Straße 32, 51067 Köln, Deutschland

**Keywords:** Geschichte der Urologie, Geschichte der Sexualmedizin, Max Marcuse, Emigration jüdischer Gelehrter, Holocaust, History of urology, History of sexual medicine, Max Marcuse, Emigration of Jewish scientists, Holocaust

## Abstract

Die Sexualmedizin entwickelte sich im deutschsprachigen Raum im letzten Viertel des langen 19. Jahrhunderts entlang einer Grenzlinie zu den klinischen Fächern Urologie, Venerologie, Frauenheilkunde, Neurologie/Psychiatrie und Innere Medizin, woraus sich vielfältige Befruchtungen, Verflechtungen, Abgrenzungsbemühungen und Überschneidungen ergaben. Wir konnten hier als einen weiteren frühen Protagonisten Max Marcuse evaluieren, der als einziger der besonders bekannten Berliner Sexualwissenschaftler Magnus Hirschfeld, Ivan Bloch und Albert Moll den Holocaust überlebte und dessen 60. Todestag in diesem Jahr ist.

## Abstract (en)

The development of sexual medicine starts in Europe in parallel to the evolving clinical specialties urology, venerology, gynecology, neurology/psychiatry, and internal medicine at the end of the 19th century in Berlin. For this reason, we find many examples of fruitful collaboration but also in segregation from each other in defining the new specialties. Max Marcuse, the only one of the well-known Berlin specialists Ivan Bloch, Magnus Hirschfeld, and Albert Moll to survive the Holocaust, was able to publish articles in Palestine and Israel from the 1930s to the 1960s. This year is the 60th anniversary of his death.

„Und niemals kann der Zeugungswille aus dem Geschlechtstrieb werden“

## Einleitung

Häufig wird die lange vergessene und auch schwierige Entwicklung der deutschen Sexualwissenschaft und Sexualmedizin mit dem Berliner Institut von Magnus Hirschfeld (1868–1935) und seinen vielfältigen Aktivitäten in Verbindung gebracht [3]. Hierbei geraten andere Entwicklungsstränge häufig in Vergessenheit, insbesondere die Verbindungen zu den sich parallel entwickelnden klinischen Fächern wie der Urologie, der Gynäkologie oder der Neurologie/Psychiatrie. In den letzten Jahren hat sich der Forschungsdiskurs deutlich verbreitert und Fahrt aufgenommen [4].

Schon detailliertere Einzeluntersuchungen allein zu weiteren Berliner Forschern wie Iwan Bloch (1872–1922), Albert Moll (1862–1939) oder auch Max Marcuse sind rar und beleuchten häufig nur einzelne Facetten ihres weit gespannten, differenzierten Oeuvre. Im „Personenlexikon der Sexualforschung“ von Sigusch aus dem Jahre 2009 steht eher die Einordnung in rein sexualwissenschaftliche Diskurse im Vordergrund. In seiner „Geschichte der Sexualwissenschaft“ sieht der gleiche Autor die Sexualwissenschaft aus der Abgrenzung zu den medizinischen klinischen Fächern wie der Urologie/Andrologie und Gynäkologie entstanden und nicht als beiderseitig befruchtende und konstituierende Wissenschaften, wobei viele Sexualwissenschaftler wie Albert Moll oder auch Magnus Hirschfeld in rein urologischen Zeitschriften Einzelpublikationen zu sexualwissenschaftlichen Themen im Grenzgebiet zur Urologie veröffentlichten.

Weiterhin muss man bedenken, dass die „reinen“ Sexualwissenschaftler gerade bei ihren privaten Patienten immer im Mitbewerb von Klinikern wie Urologen, Frauenärzten oder Psychiatern standen. Das waren die eigentlichen Fachgebiete, bei denen die Sexualorgane in den eigenen Fachkanon integriert waren und sind – die aber gerade nicht den Begriff „Sex“ im Namen tragen, was für Patienten bis zum heutigen Tage vielfach angenehmer und weniger stigmatisierend zu sein scheint. Weitere Fachrepräsentanten sahen ihre eigene klinische Tätigkeit mehr in der Nähe zu Neurologie – Psychologie-Psychiatrie wie Albert Moll, der auf seinem Briefkopf – „Institut für praktische Psychologie“ hervorhob, um nicht den Begriff „Sex“ zu annoncieren.

Max Marcuse war der jüngste der vier fachprägenden Berliner Repräsentanten dieser Ära, zu denen auch, nicht nur in der urologischen Erinnerungskultur häufig vergessen, Gottfried Benn (1886–1956) oder Hans Haustein (1894–1933)^1^ gehörten.

Viele frühe Sexualforscher waren als Privatgelehrte ohne zweiten akademischen Grad und damit ohne stärkere Verbindungen beispielsweise an die Berliner Friedrich-Wilhelms-Universität in eigener, oft umsatzstarker Praxis im Großstadtbereich tätig, was ihnen eine unabhängige wissenschaftliche Tätigkeit erst ermöglichte. Weiterhin waren aufgrund persönlicher Gegensätze die Kommunikation der einzelnen Protagonisten untereinander häufig erschwert. Diese entzündeten sich vielfach an der Frage „reine Wissenschaftlichkeit versus gesellschaftliche Wirksamkeit“, die bis heute in der Sexualwissenschaft ein innewohnendes Movens ist.

Die allgemeinen Lexikaeinträge repetieren bei Max Marcuse bis heute einen Normalfaktenstand, der über eine unter Rolf Winau (1937–2006) betreute Dissertation von Thomas Mayer nicht hinausgeht.

Wir wollen versuchen, das vielschichtige, formal mehreren wissenschaftlichen Fachbereichen angehörende, interdisziplinäre Werk Max Marcuses und dessen Ausstrahlung in die jeweiligen medizinischen Fächer einzuordnen, insbesondere, da bei der parallelen Fachspezialisierung sowohl von Urologie als auch von Sexualmedizin während der 1920er-Jahre durchaus verschiedene Varianten wissenschaftlicher und praktischer urologischer/venerologischer/sexualmedizinischer Tätigkeit für einen niedergelassenen Arzt ohne direkte Universitätsaffiliation besonders im Großstadtbereich möglich waren.

## Kurze Vita

**Figure 1:**
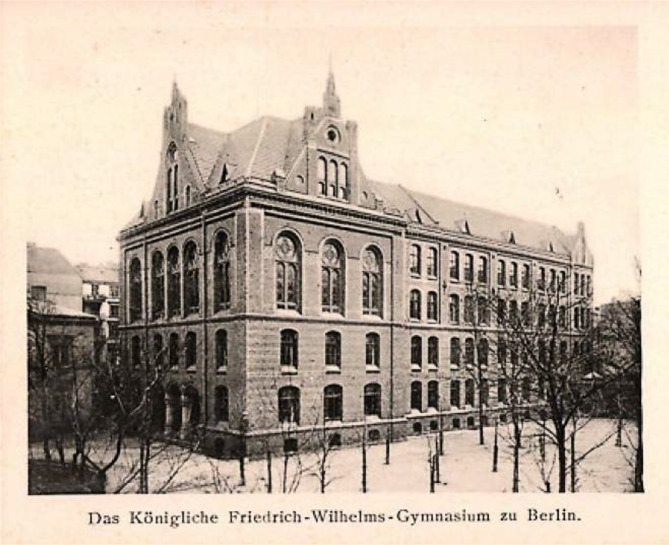

Max Marcuse wurde am 14. April 1877 in Berlin in ein jüdisch stämmiges Elternhaus geboren, das in der Neumark (poln. Nowa Marchia) nordöstlich der Oder, bis 1945 zur preußischen Provinz Brandenburg (Regierungsbezirk Frankfurt) bzw. der nordöstlichste Teil zur Provinz Pommern gehörig, seine Wurzeln hatte. Sein Vater Carl (1831 Schwerin–1906 Berlin) war Kaufmann. Für das Jahr 1880 lässt sich ein C. Marcuse in der Dragonerstraße für Berlin nachweisen.^2^ Seine Mutter Johanna, geborene Labus (1840–1912), entstammte einer dortigen Mühlenbesitzerfamilie. Er wuchs somit in einem gutbürgerlichen assimilierten, finanziell gut situierten Umfeld auf. Er hatte zwei Schwestern (Hedwig M 1861–1875) und (Lina M 1864–1938). Max Marcuse besuchte das Sophiengymnasium in der Spandauer Vorstadt bis zur „Befähigung zum einjährigen Militärdienst“ in Berlin und später das renommierte königliche Friedrich-Wilhelms-Gymnasium, an dem er „zu Michaelis“ 1895 seine Reifeprüfung ablegte. Das Königliche Friedrich-Wilhelms-Gymnasium erfreute sich regen Zuspruchs der Berliner Eliten und erreichte vor der Jahrhundertwende mit fast 1000 Schülern zumeist aus protestantischen und jüdischen Familien hohe Schülerzahlen (Abb. 1).

### Infobox Schüler des Friedrich-Wilhelms Gymnasiums Berlin

- James Israel 1848–1926 Urologe
- Max Dessoir 1867–1947 Arzt
- Walther Rathenau 1867–1922 Politiker
- Johannes Sobotta 1869–1945 Anatom
- Arthur Pappenheim 1870–1916 Internist

**Figure 2:**
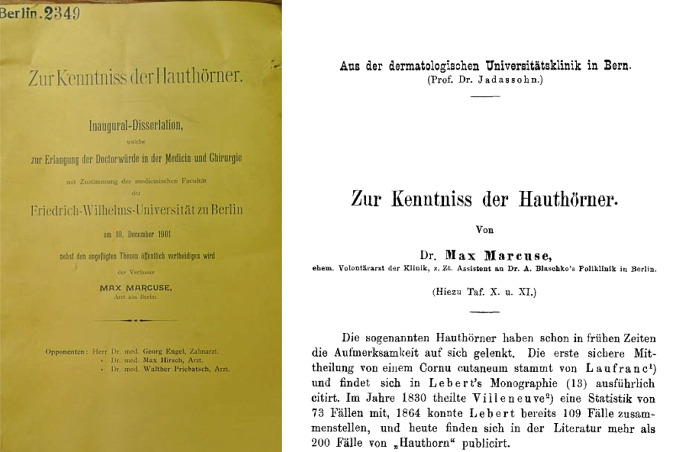

**Figure 3:**
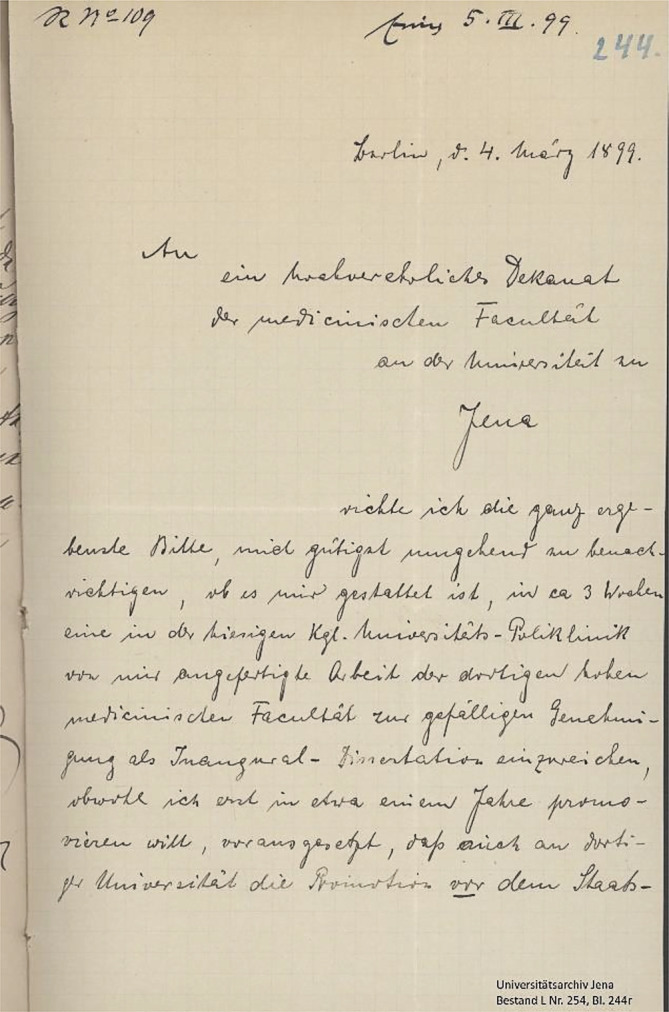

Danach begann er das Studium der Medizin in Berlin (1895–1898) an der Friedrich-Wilhelms-Universität und hörte Vorlesungen u. a. bei dem Zoologen Oskar Hertwig (1849–1922), den beiden Internisten Georg Klemperer (1865–1946 Boston) und Hermann Senator (1834–1911), dem berühmten Pathologen Rudolf Virchow (1821–1902), dem Anatomen Wilhelm Waldeyer (1836–1921) und dem Neurologen und Psychiater Emanuel Mendel (1839–1907).^3^ Zeittypisch wechselte Marcuse mehrfach den Studienort und verbrachte das Sommersemester 1898 in Würzburg^4^ und einige Zeit in Freiburg/B. (1898–1900). Sein Medizinische Staatsexamen legte er am 1. Juli 1900 in Freiburg mit der Note „gut“ ab und erhielt seine Approbation am 9. Juli 1900. Er promovierte im Jahre 1901 (Colloquium 12. November 1901) in Berlin mit einem dermatologischen Thema bei „Zur Kenntnis der Hauthörner“^5^ (Abb. 2). Bereits im März 1899 hatte Marcuse an der Medizinischen Fakultät in Jena angefragt, ob er eine in Berlin angefertigte Arbeit in Jena als Dissertationsschrift einreichen könne, in der Hoffnung, in Jena bereits vor dem medizinischen Staatsexamen promoviert werden zu können. Noch immer galt zu dieser Zeit die Promotion als universitärer Abschluss des Medizinstudiums, obwohl in Preußen das Staatsexamen als medizinische Endprüfung des Staates mit konsekutiver Approbation bereits ab 1825 eingeführt war. Gerade zu dieser Zeit hatte die Reformdiskussion, die 1901 in einer großen Studienreform mündete, deutlich wieder an Fahrt aufgenommen. Die Fakultät lehnte – nachdem zunächst eine Bescheinigung des Direktors der medizinischen Poliklinik über den Sachverhalt eingefordert wurde – das Gesuch ab, da die Zulassung zur Promotionsprüfung nach Jenenser Regeln ein mindestens 8‑semestriges Studium voraussetzte, was Marcuse zu dieser Zeit noch nicht vorweisen konnte^6^ (Abb. 3).

Nach dem Staatexamen war er zunächst „Volontär – Assistent“ bei dem Neisser Schüler Josef Jodassohn (1863–1936) in Bern. Jadassohn beschäftigte sich in diesem Grenzgebiet besonders mit Fragen der Prostitution und der Verhütung von Geschlechtskrankheiten. Nach Ablegen seiner Promotion (Berlin) Ende 1901 arbeitete Max Marcuse bis Sommer 1902 in der privaten, nicht universitären Poliklinik für Haut- und Geschlechtskrankheiten von Alfred Blaschko (1858–1922)^7^, dem Mitbegründer der deutschen Gesellschaft zur Bekämpfung der Geschlechtskrankheiten 1902 in Berlin. Hier erhielt Marcuse sicherlich die fachliche Prägung zu den Wissenszusammenhängen von Geschlechtskrankheiten und Prostitution, die sein Mitstreiter Iwan Bloch als ein wichtiges konstituierendes Merkmal der sich entwickelnden neuen Spezialdisziplin Sexualwissenschaft herausstellte. Gleichzeitig war das Themenfeld Geschlechtskrankheiten mit ihren Auswirkungen auf den Harntrakt (gonorrhoische Urethralstrikturen – syphilitische Blasen- und Nierenveränderungen) für die sich parallel entwickelnden Urologie fachbildend.

Diese für Marcuse wichtige Prägung wird dadurch unterstrichen, dass Blaschko in der von Marcuse herausgegebenen Zeitschrift *Sexual-Probleme* als ständiger Mitarbeiter im Frontispiz aufgeführt wurde.

**Figure 4:**
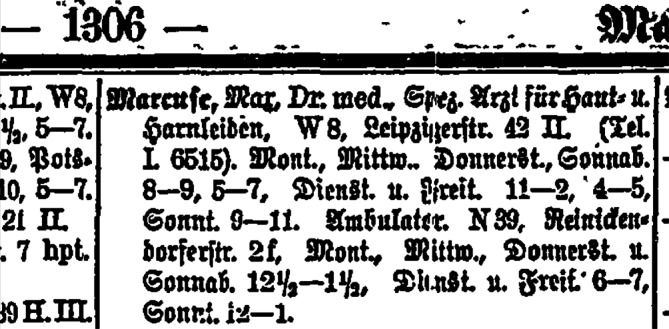

Ab September 1902 war Max Marcuse besoldeter „Hülfsarzt“ in der Hautkranken-Station des Frankfurter Städtischen Krankenhauses^8^, das von Karl Herxheimer (1861–1942) geleitet wurde. Im Februar 1903 verließ Hans Marcuse das Krankenhaus, nachdem er bei der Besetzung einer Sekundärarztstelle zugunsten eines Stadtratssohns übergangen worden war.^9^ Danach ließ sich er sich im Jahre 1904/05 als „Arzt für Haut- und Harnleiden“ in Berlin in der stark frequentierten Leipziger Straße nieder (Abb. 4, 5 und 6).

**Figure 5:**
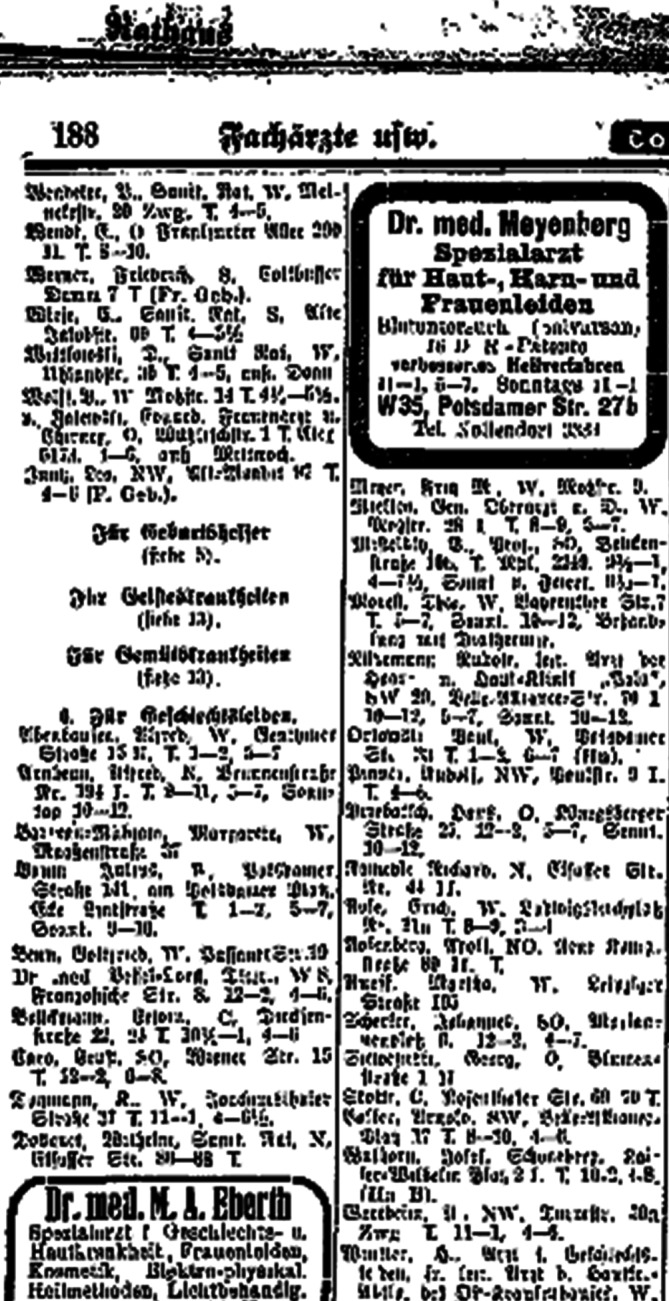

**Figure 6:**
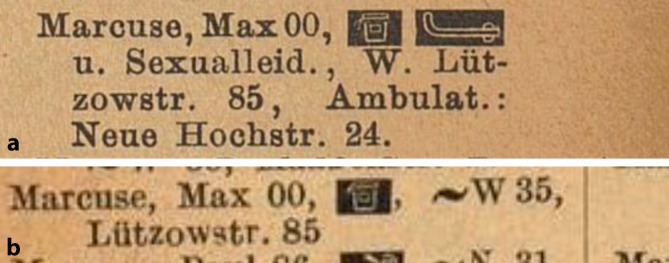

**Figure 7:**
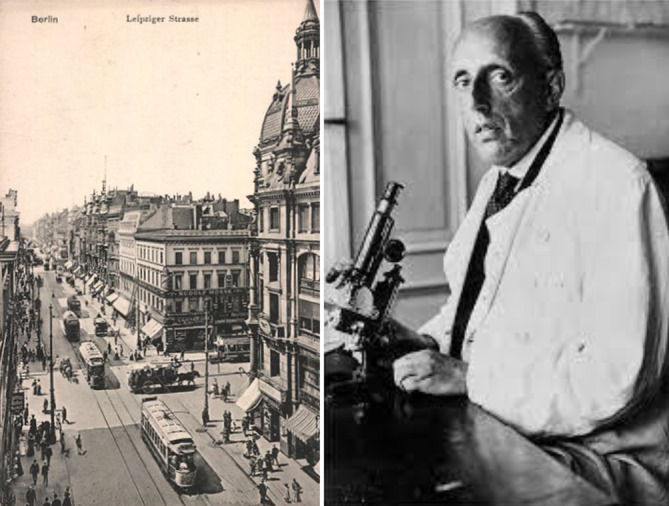

Max Marcuse heiratete 1905 die 3 Jahre jüngere, in Berlin geborene Mühlenbesitzertochter Helene Frida Elisabeth Kohls (1880–1961), die einen Teil ihrer Kindheit in Althöfchen in der Neumark (Stary Dworek pol., Landkreis Schwerin an der Warthe, Provinz Posen) verbracht hatte. Von dieser ließ er sich nach mehr als 20 Jahren scheiden.^11^,^12^ Aus dieser Ehe entstammte der Sohn Hans Renatus (Yohanan Meroz 1920–2006), der später Botschafter Israels in der Bundesrepublik war. 1936 heiratete Max Marcuse in Palästina Grete Seelenfreund geb. Freudenthal (–1984), mit der er schon seit 1931 einen Sohn (Michael; hatte Abb. 7).

Bereits im Sommer 1933 fasste Max Marcuse den Entschluss, aufgrund der politischen Lage nach Palästina zu emigrieren (Tab. 1).

**Table 1:** 

1. April 1933	„Judenboykott“, „Kauft nicht bei Juden“
07.04.1933	„Gesetz zur Wiederherstellung des Berufsbeamtentums“. Juden werden durch den „Arierparagraph“ vom Beamtenberuf ausgeschlossen. In den folgenden Wochen wird der Arierparagraph in zahlreichen Berufen eingeführt (z. B. Ärzte, Rechtsanwälte, Notare, Behördenangestellte, Mitglieder wissenschaftlicher Vereinigungen)
22.04.1933	„Verordnung über die Zulassung von Ärzten zur Tätigkeit bei den Krankenkassen“, de facto Berufsverbot für jüdische Kassenärzte
25. April 1933	„Gesetz über die Überfüllung deutscher Hochschulen“. Die Zahl der Neuaufnahmen jüdischer Studenten an allen deutschen Hochschulen wird auf 1,5 % beschränkt, der Gesamtanteil auf 5 %
10.05.1933	„Bücherverbrennung“ „Wider den undeutschen Geist“
14. Juli 1933	„Gesetz über den Widerruf von Einbürgerungen und die Aberkennung der deutschen Staatsangehörigkeit“. Deutschen Reichsangehörigen, die sich im Ausland aufhielten und dort durch ihr Verhalten „gegen die Pflicht zur Treue gegen Reich und Volk“ verstießen und die „deutschen Belange“ schädigten, konnte die Staatsangehörigkeit entzogen werden. Auch Personen, die einer Aufforderung zur Rückkehr nicht nachkamen, konnte die deutsche Staatsangehörigkeit entzogen werden. Die nationalsozialistischen Machthaber konnten sich die von Juden zurückgelassenen Vermögen mit scheinbarer Legalität aneignen, indem sie ein Verfahren einleiteten, das mit der Aberkennung der deutschen Staatsangehörigkeit und – damit verbunden – dem Vermögenseinzug endete

Mit der Bahn gelangte er mit seinem Sohn nach Triest, von dort mit dem Dampfer „Martha Washington“ der „Austro-Americana-Linie“, einer österreich-ungarischen Reederei, nach Palästina.

**Figure 8:**
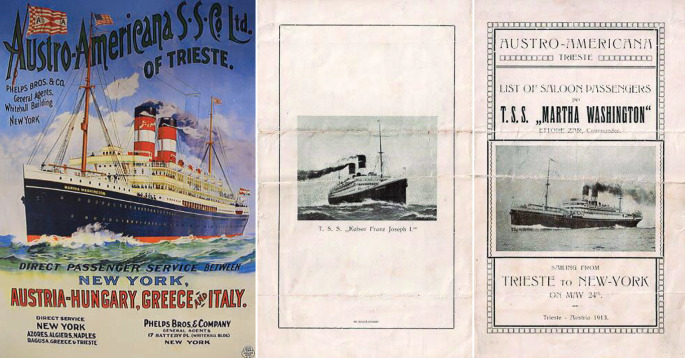

In den Jahren 1933 bis 1936 war Palästina das wichtigste Exilland für jüdische Flüchtlinge. 1933 emigrieren etwa 38.000 Juden von insgesamt 525.000 jüdischen oder dem Judentum nahestehenden Mitbürgern des Deutschen Reiches. Am 3. August 1933 erreichte Hans Marcuse den Hafen von Jaffa. Auf der Reise ging ein von Lovis Corinth (1858–1925) angefertigtes Portrait Marcuses verloren (Abb. 8).

Hier in Israel konnte Max Marcuse, wie viele andere, nicht mehr (nicht nur aufgrund von Sprachproblemen) an seine alten Erfolge anknüpfen. Dies war ein Problem, was bei vielen in Deutschland vollständig assimilierten Juden bestand.

Max Marcuse verstarb in Tel Aviv am 24.06.1963.

## Wissenschaft

**Figure 9:**
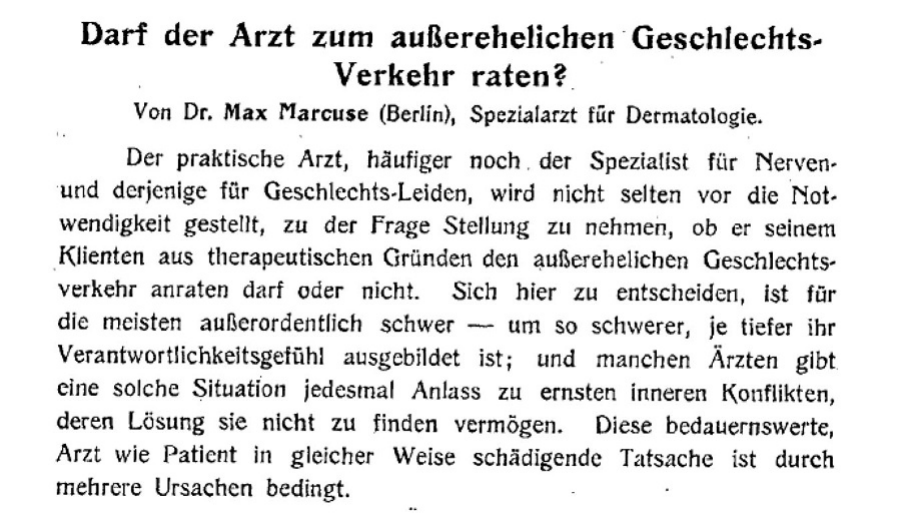

Bereits im Studium war Marcuse mit den Problemen von unehelichen Müttern und Abtreibung nach Zeitzeugenangaben in Berührung gekommen. Dieses Problem des außerehelichen Geschlechtsverkehrs prägte seine wissenschaftlichen Arbeiten wesentlich. Hieraus resultierten bereits frühe Zeitschriften- und Buchpublikation. Zuvor hatte er, venerodermatologisch geprägt, 1907 die Buchpublikation ,Hautkrankheiten und Sexualität‘ verfasst sowie Buchbesprechungen in der Fachliteratur, die ihn als besonderen Kenner der Materie ausweisen. Während seiner klinischen Zeit verfasst er eigene Arbeiten und Artikelrezensionen in einer renommierten venerodermatologischen Fachzeitschrift, dem bei Julius Springer herausgegebenen und bis heute bestehenden *Archiv für Dermatologie und Syphilis*. Auch war er allgemeinbildend publizistisch tätig (Abb. 9 und 10).

**Figure 10:**
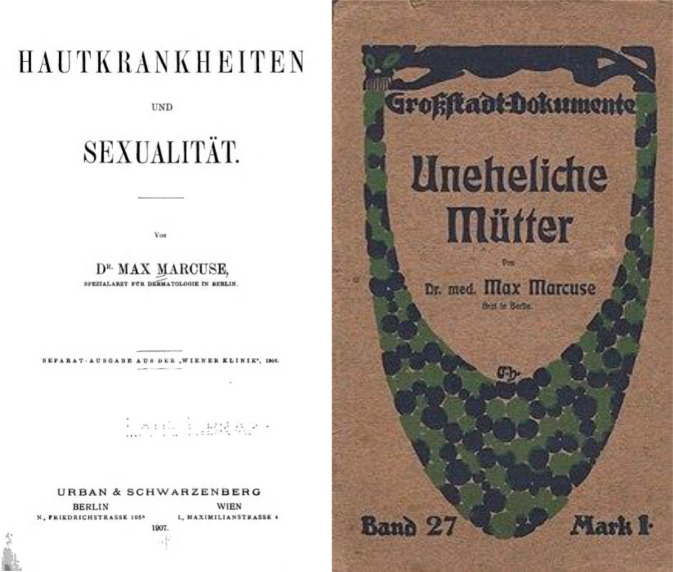

Im Kreise der Sexualforscher war Marcuse entscheidend bei Diskursen zur Heterosexualität beteiligt und stand den Diskursen zur Homosexualität ablehnend gegenüber. Für ihn war der zentrale Ansatzpunkt der Sexualreform nicht die Erforschung des „Perversen“, sondern die Modernisierung der Heterosexualität.

**Figure 11:**
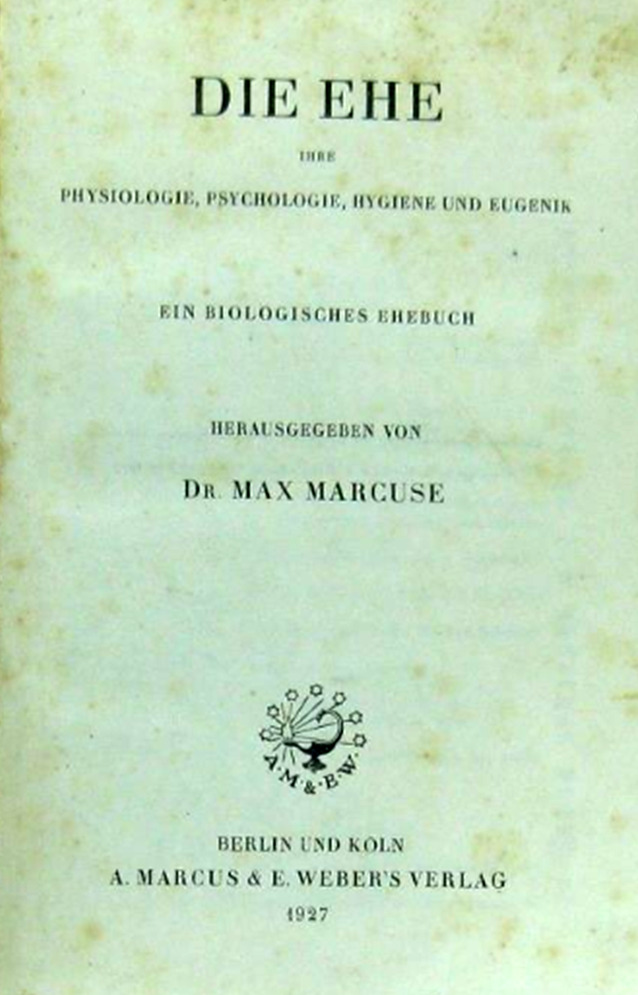

Er gehörte zu der Vielzahl von Autoren der Zeit, die Ehehandbücher verfassten oder sich mit Beratungsfragen beschäftigten und sich in dieser Publikationsform an ein akademisches Publikum wandten, indem arrivierte Wissenschaftler wie z. B. Albert Moll, der nicht unumstritten war, gewonnen wurden (Abb. 11).

Mit dieser Arbeit forderte er insbesondere die katholische Moraltheologie heraus. Denn Marcuse vertrat die Ansicht, dass Ärzte und kein Priester für die Ehevorbereitung zuständig sein sollten. Darüber hinaus war Marcuse ein engagierter Streiter für eine detaillierte Vorbereitung auf die Ehe, worunter er neben einer umfänglichen Unterrichtung über Geschlechtskrankheiten auch die Vermeidung jeder sexuellen Abstinenz verstand. Diese benannte er als Hauptursache für zahllose psychosomatische Leiden und die damals als allgemein verbreitete Zivilisationskrankheit benannte „Nervosität“. Als er immer mehr in kirchlichen Zeitschriften und auf Tagungen attackiert wurde, griff Marcuse zum äußersten Mittel: Er veröffentlichte die Fallstudie eines Patienten, der katholischer Priester war und schilderte bis ins letzte Detail dessen sexuelle Phantasien, die Folge des Zölibats seien. Daraufhin verstummten seine Gegner schlagartig und bedachten ihn für die nächsten Jahrzehnte mit Schweigen.

## Urologie

Im Gegensatz zu weiteren wichtigen Protagonisten der frühen Sexualwissenschaft widmete sich Max Marcuse wie auch Hermann Rohleder (1866–1934) in Leipzig oder Samuel Jessner (1859–1929) in Königsberg diesem besonderen interdisziplinären Arbeitsfeld, was aus der eigenen klinisch-praktischen Arbeit in niedergelassener Praxis resultierte. Die Arbeiten sind ein wichtiges Zeugnis und zugleich frühe Quellen der frühen Verbindung von Sexualmedizin mit der Urologie und Venerologie. Marcuse schaffte es, diese Themen in allgemeinen medizinischen Zeitschriften zu publizierten. Daher ist er, wie andere, die meist nur in den jeweiligen fachspezifischen Blättern publizierten, in der urologischen Erinnerungskultur oder der von Venerologen oder Dermatologen wenig verankert.

Auch war der „Broterwerb“ in einer umsatzstarken Praxis notwendig, um als Privatgelehrter ohne Verbindung zu einer Universität oder Forschungseinrichtung arbeiten zu können. Dies wird vielfach in der Historiographie übersehen, wenn auf die Spezialisierung als Sexualwissenschaftler abgehoben wird. Bisher wurde nur die finanzielle Situation des Hirschfeld-Instituts beleuchtet. Die Behandlung von Geschlechtskrankheiten, die in der vorantibiotischen Ära bis 1945 aufwendig war und beispielsweise bei der Gonorrhö Instillationsschemata z. B. nach Janet oder Harnröhrenbougierungen in aufsteigender Durchmesserfolge (Dilatatoren nach Kollmann^13^ oder Oberländer) vorsah, war beispielsweise in der Preugo (Preußische Gebührenordnung 1896 eingeführt als Ablösung der Medizinaltaxe von 1815, bis 1965 gültig)^14^ und der Adgo (Allgemeine Deutsche Gebührenordnung der Ersatzkassen 1924–1982)^15^ durchaus gut dotiert, aber nicht auf der Höhe einer blinden Blasensteinlithotripsie. Auch die Salvarsan-Therapie ab 1910/1911 wie auch die zuvor durchgeführten „Schmierkuren“ zur Quecksilberanwendung erforderten mehrfaches Erscheinen der Patienten. Somit bot diese spezialisierte Beschäftigung im Großstadtbereich ein finanziell auskömmliches und zugleich gesichertes Einkommen, da die Patienten die Diskretion einer Fachpraxis, die häufig unter einem anderen Label annoncierte, zu schätzen wussten, besonders, wenn deren Inhaber auch publizistisch tätig waren.

Hans Haustein (1894–1933)^16^, der in Berlin Wilmersdorf eine ähnliche Praxis betrieb und sich auch wissenschaftlich auf ähnlichem Gebiet betätigte, gab für das Jahr 1900 allein für Berlin 8529 Männer an, die geschlechtskrank in der Behandlung von Ärzten standen, in 107 preußischen Städten waren im Jahre 1900 23,7 % der männlichen und 25,5 % der weiblichen Bevölkerung an Geschlechtskrankheiten erkrankt.

**Figure 12:**
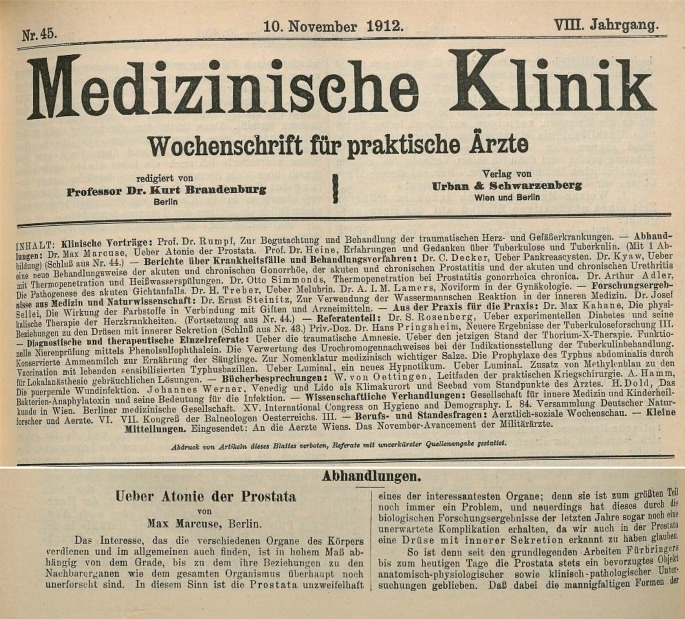

Im Jahre 1912 schrieb Max in der viel gelesenen *Medizinischen Klinik* über die „Atonie der Prostata“ (Abb. 12).

Weitere reine urologische Themen waren „Orgasmus ohne Ejakulation“ im Jahre 1922 in der renommierten *Deutschen Medizinischen Wochenschrift* sowie eine Arbeit über die Enuresis nocturna als sexualneurotisches Problem in der *Zeitschrift für Sexualwissenschaft und Sexualpolitik* 1924.

In seiner Arbeit zur „Zur Kenntnis des Climacterium virile, insbesondere über urosexuelle Störungen und Veränderungen der Prostata bei ihm“ publiziert er in Nachfolge Freuds zu den hormonellen und psychologischen Störungen eines beginnenden Testosteronmangels, was für eines Teils seines Klientel zeittypisch war, wobei er neben sexuellen Abirrungen eine bestehende Oligospermie herausstellte.

**Figure 13:**
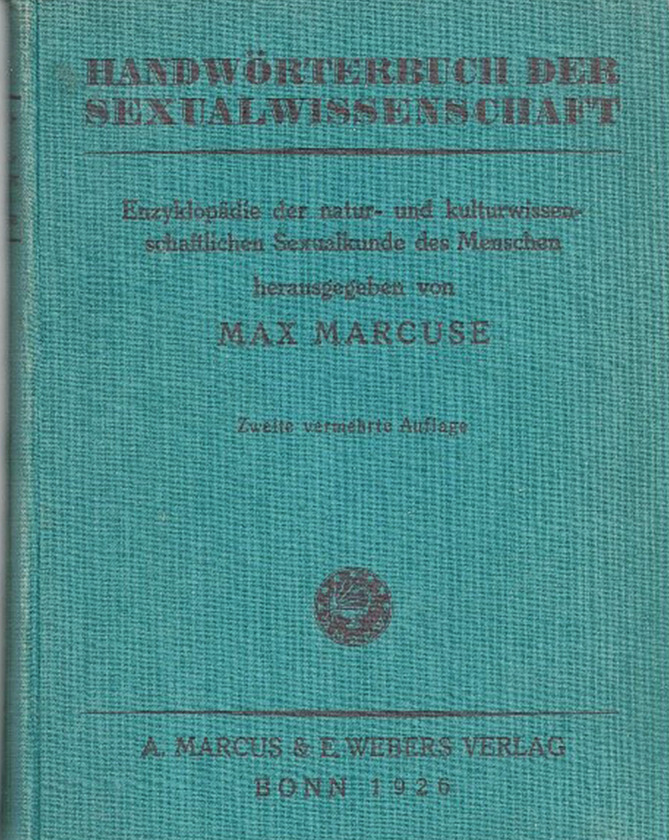

Mit Bezug zur Urologie verfasste er in seinem eigenen Handwörterbuch der Sexualwissenschaft 1923 die Stichworte Enuresis, Kastration, Klimakterium des Mannes, Pubertät. Der Wiener Oskar Scheuer (1876–1941 Ghetto Litzmannstadt [Lodz, Polen]), der für das dortige von Leo Schidrowitz (1894–1956) inaugurierte „Wiener Institut für Sexualforschung“ tätig war, verfasste weitere urologischen Stichworte für diese Publikation wie Aphrodisiaka, Beschneidung, Priapismus (Abb. 13).

**Figure 14:**
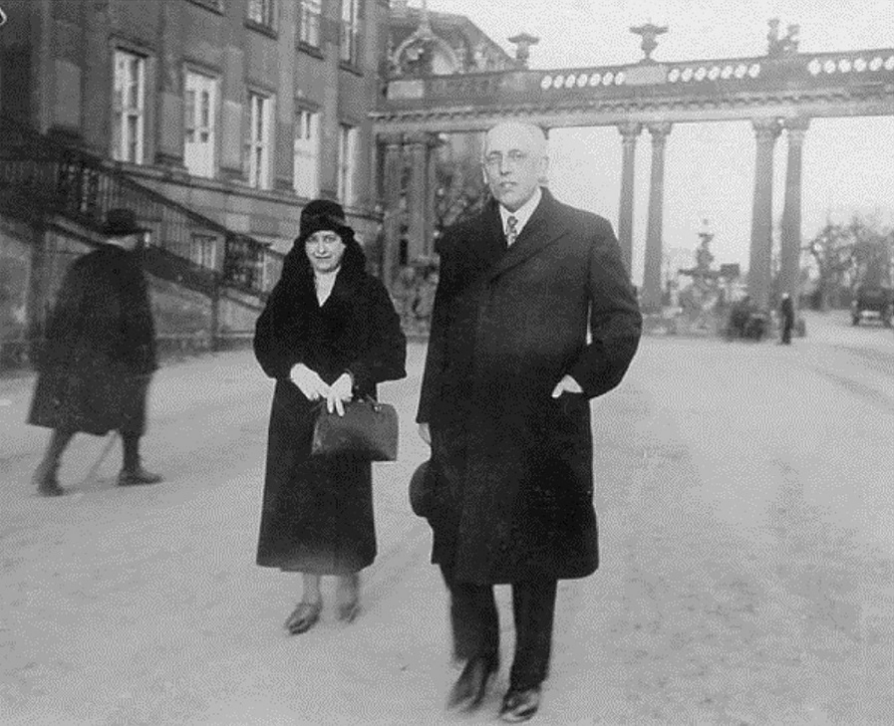

Im Handbuch der Sexualwissenschaften von Albert Moll bearbeitete Max Marcuse das Stichwort „Neuropathia sexualis“ (Abb. 14).

## Zur Verhütungsfrage – Eugenik

Für manche ist die Tatsache befremdlich, dass Max Marcuse – wie viele Forscher seiner Zeit – sich einerseits mit für uns noch immer aktuellen Fragen beschäftigen konnte, andererseits aber auch Fragen der Eugenik – ähnlich Helene Stöcker oder Magnus Hirschfeld – durchaus positiv gegenüberstand. Das waren Debatten, die auch Urologen wie Friedrich Wilhelm Schallmayer (1857–1919), der länger in Düsseldorf wirkte, maßgeblich prägten und zu dem er sich publizistisch äußerte. Marcuse schätzte besonders den Rassebiologen und Polygynisten Christian Freiherr von Ehrenfels (1859–1932), dem er in Publikationen immer wieder zustimmte. Ehrenfels war ebenso wie Marcuse ein engagierter Gegner kirchlichen Einflusses auf das Sexualleben der Menschen.

**Figure 15:**
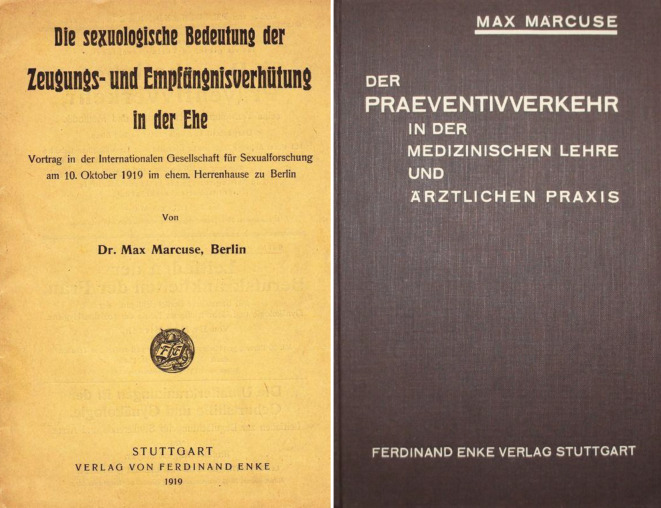

Marcuse setzte er sich für die Verhütung beim Geschlechtsverkehr in Wort und Schrift vehement ein – ein Thema, das zu dieser Zeit noch keine Breitenwirkung besaß (Abb. 15).

Insgesamt erschienen von ihm mehr als 70 Originalarbeiten (Buchpublikationen und wissenschaftliche Artikel) sowie über 400 Rezensionen in den von ihm redigierten Zeitschriften.

## Verbandspolitik und Netzwerke

Im Jahre 1904/1905 war Max Marcuse, zu dieser Zeit 27 Jahre alt, Mitbegründer des Bundes für Mutterschutz (Helene Stöcker, 1869–1943, New York) und Ausschussmitglied. Er leitete von seiner Berliner Praxis aus zunächst die Geschäfte.

Im Jahre 1913 war er Mitbegründer der „Internationalen Gesellschaft für Sexualforschung“ (IGSF) zusammen mit Albert Moll, um gegen die bereits existierende „Ärztliche Gesellschaft für Sexualwissenschaft und Eugenik“ (ÄGESE), in der Albert Eulenburg, Iwan Bloch und Magnus Hirschfeld prägend waren und der auch der Urologe Carl Posner (1854–1928) angehörte, eine Organisation in Stellung zu bringen, die die Sexualforschung „rein“ wissenschaftlich, vollkommen unparteiisch und insbesondere über den medizinischen Tellerrand hinausblickend betreiben sollte.

**Figure 16:**
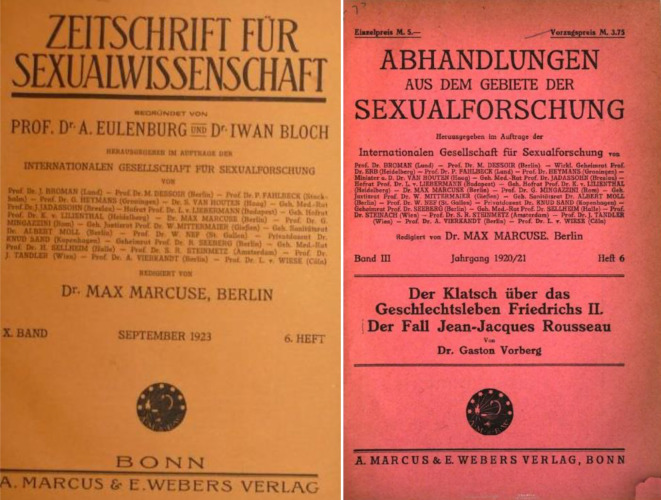

Von 1908 bis 1914 gab Max Marcuse die *Sexual-Probleme – Zeitschrift für Sexualwissenschaft und Sexualpolitik*, Sauerländer Verlag, heraus. Von 1919–1932 die *Zeitschrift für Sexualwissenschaft* sowie von 1918–1931 redigierte er die Monographiereihe „Abhandlungen aus dem Gebiete der Sexualforschung“ (Abb. 16).

Ebenfalls redigierte er die Verhandlungen des I. Internationalen Kongresses für Sexualforschung, Berlin, vom 10. bis 16. Oktober 1926.

**Figure 17:**
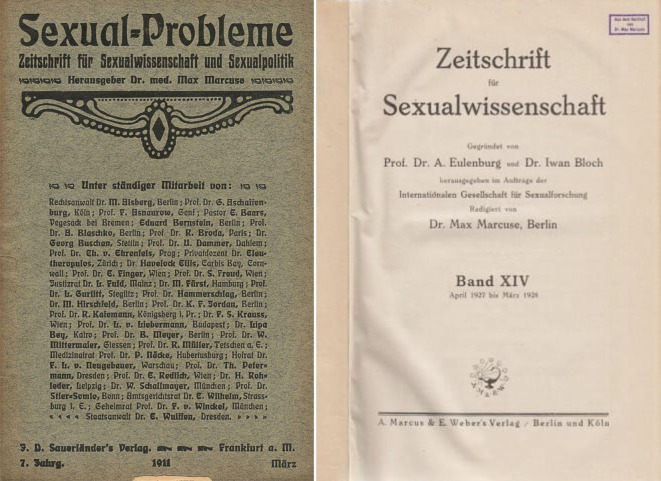

Allein diese Tätigkeiten zeigen seine gute Integration in die Gruppe der Berliner und internationalen Sexualmediziner/-wissenschaftler, die aber auch nicht immer frei von Spannungen war. Mit Helene Stöcker und den tonangebenden Frauen des Bundes für Mutterschutz überwarf sich Marcuse theoretisch, politisch und persönlich in der Frage der Herausgeberschaft der *Zeitschrift für Mutterschutz* Ende 1907. Im Kern ging es bei der Trennung um eine Unvereinbarkeit von „objektiv“-wissenschaftlichem Anspruch einerseits, den er vertrat und politisch-„fürsorglicher“ Reformarbeit andererseits, für die im Bund v. a. Helene Stöcker und Maria Lischnewska (1854–1938) standen. Die Herausgeberarbeit in der Zeitschrift *Mutterschutz – Zeitschrift zur Reform der sexuellen Ethik* riss er an sich und nannte sie in *Sexual-Probleme* um und vereinigte diese 1909 mit der initial von Eulenburg und Bloch 1908 begründeten *Zeitschrift für Sexualwissenschaft* (Abb. 17).

Max Marcuse war nicht Mitglied der Deutschen Gesellschaft für Urologie.

## Exodus

Nach seiner Emigration im Jahre 1933 im Alter von 56 Jahren gelang es Max Marcuse aus vielfältigen Gründen nicht mehr, an seine bestehenden Netzwerke anzuknüpfen. Dies lag zum einen an sprachlichen Problemen im Hebräischen, die es ihm nicht ermöglichten, in Israel ausreichend zu kommunizieren. Auch fanden seine in der Zeit vor 1933 geprägten Auffassungen im sich neu bildenden Staat Israel mit einer gänzlich anderen Bevölkerungsstruktur und Sexualitätsauffassung keine Basis, wie Kirsten Leng kürzlich detailliert herausstellte. Aber auch seine fehlende Möglichkeit, in der englischen Sprache ausreichend zu kommunizieren, war für seine Isolation verantwortlich, obwohl er der einzige der vier großen Berliner Sexualwissenschaftler war, der nicht in den 1930er-Jahren verstorben war.

In der Zeitschrift *Der Orient*, der an die Form der *Die Weltbühne* angelegt war und in deutscher Sprache erschien, publizierte Marcuse zu sexueller Tradition im Judentum. In einer weiteren Ausgabe der Zeitschrift beschäftigt ihn das Urteil in einem Sexualprozess, das wesentlich auf dem festgestellten Charakter des Angeklagten beruhte. Hier konnte er auf eigene Publikationen aus den 1920er-Jahren zurückgreifen.

Auch zu Sexualproblemen im Kibbuz nahm er in deutscher Sprache Stellung.

**Figure 18:**
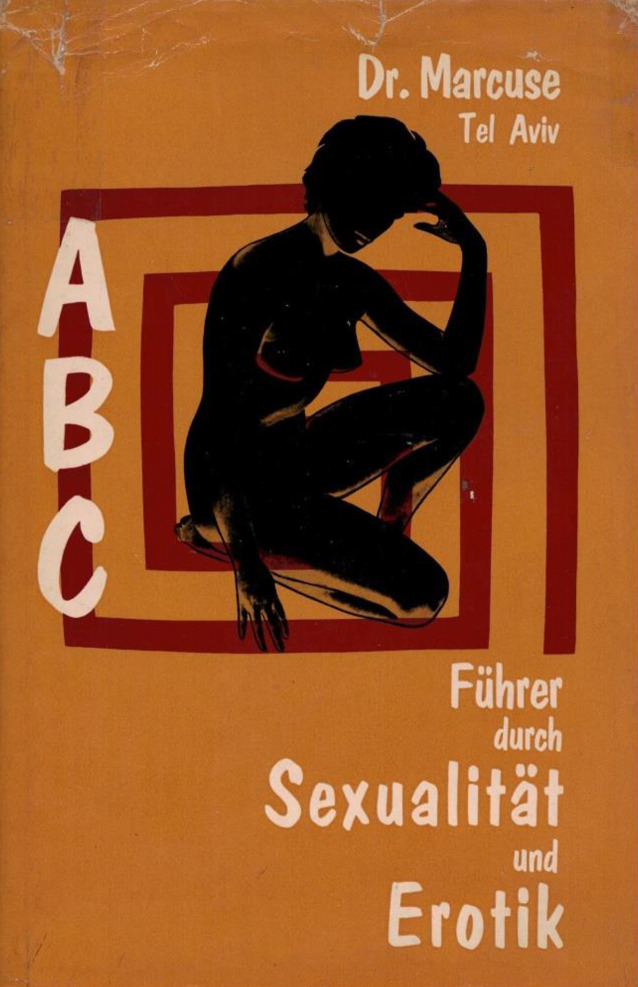

An alte Erfolge knüpft nur noch einmal in kleineren Publikationen 1948–1949 sowie in seinem „ABC Führer durch Sexualität und Erotik“ an. Dies wird von manchen Autoren als Fauxpas angesehen, da der Verlag dem Beate Uhse-Konzern nahestand. Zu diesem Buch hatte der in den 1950er- und 1960er-Jahren renommierte Hamburger Sexualforscher Hans Giese (1920–1970)^17^ das Vorwort verfasst (Abb. 18). Giese versuchte ebenfalls, Marcuse für die neu gegründete Gesellschaft für Sexualforschung zugewinnen. Hierzu war Marcuse aber nicht bereit. Er bezweifelte, dass „in einer sich als ‚deutschen‘ Gesellschaft bezeichnenden Organisation (…) fuer (sic) einen vormals deutschen Juden heute noch oder wieder ein legitimer Platz sein koennte (sic)“.^18^

## Zusammenfassung – Fazit für die Praxis

In der schwierigen Gründungsphase der deutschen Sexualmedizin/-wissenschaft spielte die Urologie in ihrer Ausprägung als Venerologie und Sexualmedizin des Mannes eine konstituierende Rolle, was sich an der Biographie Max Marcuses gut ablesen lässt, ein Momentum, das in der Erinnerungskultur sowohl der Urologie, Venerologie und Sexualmedizin verloren gegangen ist.

Das Jahr 1933 bedeute für beide Fächer, Urologie und Sexualmedizin, einen wesentlichen Einschnitt, da die vor 1933 tätigen Forscher, häufig jüdischer Herkunft, nach dem Kriege oder in der Emigration an ihre alten Erfolge und Positionen nicht mehr anknüpfen konnten (aufgrund Alters, Sprachproblemen, Verschiebung des Wissenschaftsdiskurses), was einen wesentlichen Wissensverlust und Änderung von Forschungsperspektiven nach sich zog. Auch wirkten die Netzwerke der jüngeren Generation, die sich in der NS-Zeit herausgebildet hatten, noch deutlich in der Nachkriegszeit in beiden deutschen Staaten in medizinischen Fachgesellschaften, Universitäten und in der Gesellschaft fort.

Marcuse gehörte in Tel Aviv neben dem gleichaltrigen Viktor Blum (1877–1954) in Chicago zu den wenigen der Urologie nahestehenden Emigranten jüdischer Abstammung, denen eine publizistische und ärztliche Tätigkeit in ihrer erzwungenen Emigration im Ausland überhaupt gelang.

## Footnotes

1 Eine Publikation zu Hans Haustein ist mit Matthis Krischel in Vorbereitung und als Vortrag angenommen.

2 Berliner Adreßbuch: für das Jahr 1880 unter Benutzung amtlicher Quellen. Loewenthal, Berlin S. 604 https://digital.zlb.de/viewer/image/34115512_1880/623/. Zugegriffen: 15.04.2023.

3 Archiv Humboldtuniversität Matrikel-Nr. 3349/85; 08.10.1895 bis 04.04.1898, Dissertation Berlin Med. Fak. 01, Nr. 724, E‑mail Frau Sandra Eigel von 18.04.2023.

4 Schreiben Universitätsarchiv Würzburg 10.05.2023 Dr. Markus Holtz, Leiter Universitätsarchiv: „Wir können lediglich nachweisen, dass ein Max Marcuse aus Berlin im Sommersemester 1898 an der Universität Würzburg eingeschrieben war. Dies ist dem Verzeichnis der Studierenden zu entnehmen. Weitere Dokumente zu seinem Studium befinden sich leider nicht in unseren Beständen, was darauf hindeuten könnte, dass er eingeschrieben war, ohne Kurse belegt zu haben“.

5 Dissertation Berlin Med. Fak. 01, Nr. 724.

6 UAJ Bestand L254 243–248. Wir danken dem Leiter des Universitätsarchives der Friedrich-Schiller-Universität Jena Herrn PD Dr. habil. Stefan Gerber für die rasche Aktenübermittlung an uns und die knappe Zusammenfassung des Verwaltungsvorganges.

7 1908 Titularprofessor, 1918 Geheimrat.

8 1. September 1902 bis zum 28. Februar 1903.

9 Stadtarchiv Frankfurt Personalakte Wilhelm Bornemann PA25.842 fol 32v und Personalakte Max Marcuse PA 25,199 fol20 v.

11 https://www.geni.com/people/Max-Marcuse-MD/6000000001053947636 Zugegriffen 07.07.2023.

12 Im Ersten Weltkrieg diente Max Marcuse in einem Reservelazarett in Frankfurt/O.

13 Eine Arbeit zu Arthur Kollmann erscheint im Septemberheft 2023 der Zeitschrift *Die Urologie*, 10.1007/s00120-023-02163-9.

14 Bekanntmachung betreffend den Erlass einer Gebührenordnung für Ärzte und Zahnärzte Deutscher Reichs = Anzeiger und Königlich Preussischer Staats = Anzeiger Beilage zu Nr 119 19.05.1896 online https://digi.bib.uni-mannheim.de/viewer/reichsanzeiger/film/142-8061/0245.jp2 zugegriffen 07.07.2023.

15 Allgemeine Deutsche Gebührenordnung für Ärzte vom 1. Januar 1928. (1941). Reichsgesundheitsverlag, Berlin/Wien.

16 Zu Hans Haustein ist ebenfalls eine Publikation mit Matthis Krischel in Vorbereitung.

17 NSDAP Mitgliedschaft seit 01.01.1942 Nr. https://www.memoiresdeguerre.com/2015/03/liste-des-membres-du-nsdap-g-h.html. Zugegriffen: 15.04.2023.

18 BArch RN 1134, 6 Brief Marx Marcuse an Hans Giese 20.09.1950, Marcuse trat dem Beirat der *Zeitschrift für Sexualforschung*, die 1950 gegründet wurde, nicht bei vgl. Liebeknecht M 2020 Wissen über Sex Die Gesellschaft für Sexualforschung im Spannungsfeld westdeutscher Wandlungsprozesse. Wallstein, Göttingen S 88–91.
